# Strontium and oxygen isotopes as indicators of Longobards mobility in Italy: an investigation at Povegliano Veronese

**DOI:** 10.1038/s41598-020-67480-x

**Published:** 2020-07-15

**Authors:** Guendalina Francisci, Ileana Micarelli, Paola Iacumin, Francesca Castorina, Fabio Di Vincenzo, Martina Di Matteo, Caterina Giostra, Giorgio Manzi, Mary Anne Tafuri

**Affiliations:** 1 Department of Environmental Biology, Sapienza Università di Roma, Piazzale Aldo Moro, 5, Roma, Italy; 2 Istituto Italiano di Paleontologia Umana, Piazza Ruggero Bonghi, 2, 03012 Anangni, Italy; 3 Dipartimento di Scienze Chimiche, della Vita e della Sostenibilità Ambientale, Università di Parma, Parco Area Delle Scienze, 11/a, Parma, Italy; 4 CNR, Istituto di Geologia Ambientale E Geoingegneria, c/o Dipartimento di Scienze Della Terra, Sapienza Università di Roma, Piazzale Aldo Moro, 5, Roma, Italy; 5 Dipartimento di Scienze Dell’Antichità, Sapienza Università di Roma, Piazzale Aldo Moro, 5, Roma, Italy; 6 Dipartimento di Storia, archeologia e storia dell′arte, Università Cattolica del Sacro Cuore, Largo Agostino Gemelli, 1, Milano, Italy

**Keywords:** Biogeochemistry, Environmental social sciences

## Abstract

The arrival of the Longobards in Northern Italy in 568 CE marked a period of renewed political stability in the Peninsula after the collapse of the Western Roman Empire. The trajectory of the spread of Longobards in Italy across the Alps and into the South is known from many literary sources. However, their mobility and residence patterns at a population level remain to be fully understood. Here we present a multi-isotopic analysis (^87^Sr/^86^Sr and ^18^O/^16^O) of 39 humans and 14 animals buried at the Longobard necropolis of Povegliano Veronese (VR, Italy; 6th—8th century CE), to address mode and tempo of the spread of this population in the Peninsula. The geographical location of Povegliano Veronese plays a key role: the site lies along the Via* Postumia*, which was one of the main ancient Roman roads of Northern Italy, representing an important route in post-classical Italy. The integration of isotopic data with the archaeological evidence allowed us to determine the presence of individuals from at least three different regions of origin, building a diachronic map of the dynamics of mobility of this group in northern Italy.

## Introduction

The fall of the Western Roman Empire in 476 CE was (at least in part) a consequence of the political and military instability that Rome had suffered for decades. The difficult balance between the eastern and western halves of the Empire had generated a void that was easily filled by the Germanic peoples that were familiar with the Roman system if only by virtue of their role as mercenary army (i.e. during the Gothic War (535–554) Huns, Gepids, Eruli, Longobards and Persians were hired by the Byzantines to re-conquest Italy)^1^. After the collapse, the Empire became hunting ground for hordes of German tribes: Visigoths, Vandals, Ostrogoths and Longobards to name a few.

The Longobards, unlike most Germanic peoples that raided the Peninsula after the fall, settled in Italy for several decades^2^, interacting with the locals. Between the sixth and eighth centuries CE they gave life to an independent kingdom, subdivided into numerous small dukedoms. After their arrival in 568 CE under King Alboin's leadership, they left many traces of their settlement in Northern Italy and their subsequent diffusion towards the south. With historical sources and archaeological data providing a background to our understanding of the Barbarian invasions across Europe, we are confronted with a multitude of information on general cultural phenomena but little evidence on single aspects at a population level. Here we attempt to integrate archaeological, skeletal, and biomolecular data at the Longobard necropolis of Povegliano Veronese to understand how and to what extent did the Longobard ingression in Italy changed the socio-cultural scenario of the Peninsula.

With a growing literature on skeletal, isotopic and ancient DNA investigations on Barbaric communities in Central Europe^3,4^, little has been done in Italy so far^5–10^. This represents a void in the literature, especially considering how central is the role of Italy in the transition between the Roman times and the following post-classical phases. If the decline of the Western Roman Empire represents a strong political and cultural reset, no place like Italy deserves to be investigated in the assessment of how this political shift had an impact on the local population in its interaction with the new “invading” peoples.

Where the historical sources are rich of information (i.e. the agreements between Longobard dignitaries and autochthonous authorities, or the presence of laws on marriages^11^), our understanding of the biological background of local and non-local populations with the transition to the Middle Ages is still lacking.

By means of oxygen and strontium isotopic assessments, we determined migration patterns of past Longobards at Povegliano Veronese. In fact, the measurement of strontium and oxygen isotopic ratios on skeletal remains is considered a reliable method to assess residential mobility and origin of archaeological communities because of the strong relation between the chemical composition of the tissues of humans and animals and the environments in which they lived in early and later life^12–15^. A background on strontium and oxygen isotope studies, with particular reference to the area under investigation is provided in the SI. Strontium signature for Veneto is provided in Table 1 SI.

**Table 1:** List of individuals selected for strontium and oxygen isotope analysis with main archaeological and skeletal information.

Grave	Species	Sex	Age range	Chronological Phase	Tipology	Area	^87^Sr/^86^Sr sampling	δ^18^O sampling
T2	*Homo*	ND	ND	Late	Multiple pit (MNI = 11)	H	Enamel	
T4B	*Homo*	M	30–40	Late	Multiple pit (MNI = 2)	H	Enamel	
T7	*Homo*	F	30–40	Ancient	Single pit	H	Enamel	
T9	*Homo*	ND	7 ± 2	Ancient	Single pit	H	Enamel	Enamel + bone
T17	*Homo*	M	40–50	ND	Single pit	H	Enamel	
T23	*Homo*	M	20–30	ND	Single pit	H	Enamel	Enamel + bone
T26	*Homo*	ND	8 ± 2	ND	Single pit	D	Enamel	Bone
T35	*Homo*	M	20–30	Ancient	Single pit	H	Enamel	Enamel + bone
T42	*Homo*	F	30–40	Ancient	Single pit	H	Enamel	
T45*	*Homo*	M	30–40	Ancient	Single pit	E	Enamel + soil	Enamel + bone
T86	*Homo*	ND	ND	ND	Multiple pit (MNI = 12)	E	Enamel	
T96	*Homo*	ND	ND	Ancient	Multiple pit (MNI = 7)	G	Enamel	
T213	*Homo*	M	40–50	Ancient	*Totenbrett*	H	Enamel	
T332	*Homo*	ND	4 ± 1	Ancient	Single pit	H	Enamel	
T348	*Homo*	ND	Adults	Late	Multiple pit (MNI = 4)	H	Enamel + soil	
T349	*Homo*	F	30–40	Ancient	Single pit	H	Enamel	Enamel + bone
T352	*Homo*	F	30–40	Ancient	Single pit	H	Enamel	Enamel + bone
T362	*Homo*	M	30–40	Late	Single pit	H	Enamel	
T364	*Homo*	M	40–50	Ancient	Single pit	H	Enamel	
T365	*Homo*	F	40–50	Late	Single pit	H	Enamel	Bone
T370	*Homo*	ND	15 ± 3	Ancient	Single pit	H	Enamel	Enamel + bone
T376	*Homo*	ND	4 ± 1	Ancient	Single pit	H	Enamel	
T378	*Homo*	M	40–50	Ancient	Single pit	H	Enamel	
T380	*Homo*	M	40–50	Ancient	Single pit	H	Enamel + bone	Enamel + bone
T382	*Homo*	F	40–50	Late	Single pit	H	Enamel	
T390	*Homo*	ND	11 ± 2	Ancient	Single pit	H	Enamel	
T395	*Homo*	M	40–50	Ancient	Single pit	H	Enamel	Enamel + bone
T405	*Homo*	ND	15 ± 3	Late	Single pit	H	Enamel	Bone
T413	*Homo*	M	30–40	Ancient	*Totenbrett*	H	Enamel + soil	Enamel + bone
T414	*Homo*	M	40–50	Late	Single pit	H	Enamel	
T426	*Homo*	F	40–50	Ancient	Single pit	H	Enamel + soil	Bone
T427	*Homo*	ND	3 ± 1	Ancient	Single pit	H	Enamel	
T429	*Homo*	F	40–50	Late	Single pit	H	Enamel	
T430	*Homo*	F	30–40	Late	Single pit	H	Enamel	Enamel + bone
T489	*Homo*	M	30–40	Ancient	*Totenbrett*	H	Enamel + bone	Enamel + bone
F1	*Equus caballus*				Midden		Enamel + bone	Enamel + bone
F2	*Sus domesticus*				Midden		Enamel + bone	Enamel + bone
F3	*Sus domesticus*				Midden			Enamel + bone
F4	*Bos taurus*				Midden		Enamel	Enamel + bone
F5	*Bos taurus*				Midden			Enamel + bone
F6	*Ovis* vel *Capra*				Midden		Enamel	Enamel + bone
F7	*Ovis* ve*l capra*				Midden			Enamel + bone
F8	Cervus elaphus				Midden			Bone

M = male; F = Female; ND = not determined; MNI = Minimum Number of Individuals. *excluded from data analysis, as chronologically inconsistent with the Longobard group.

### Archaeological background

The necropolis of Povegliano Veronese is located 15 km southwest of Verona, in the district called Madonna dell'Uva Secca. Eastwards, the village of Ortaia was studied by the Superintendence of Verona in 1985–1986, and further in 1992–1993, as a result of construction works in the area^16,17^.

**Figure 1:**
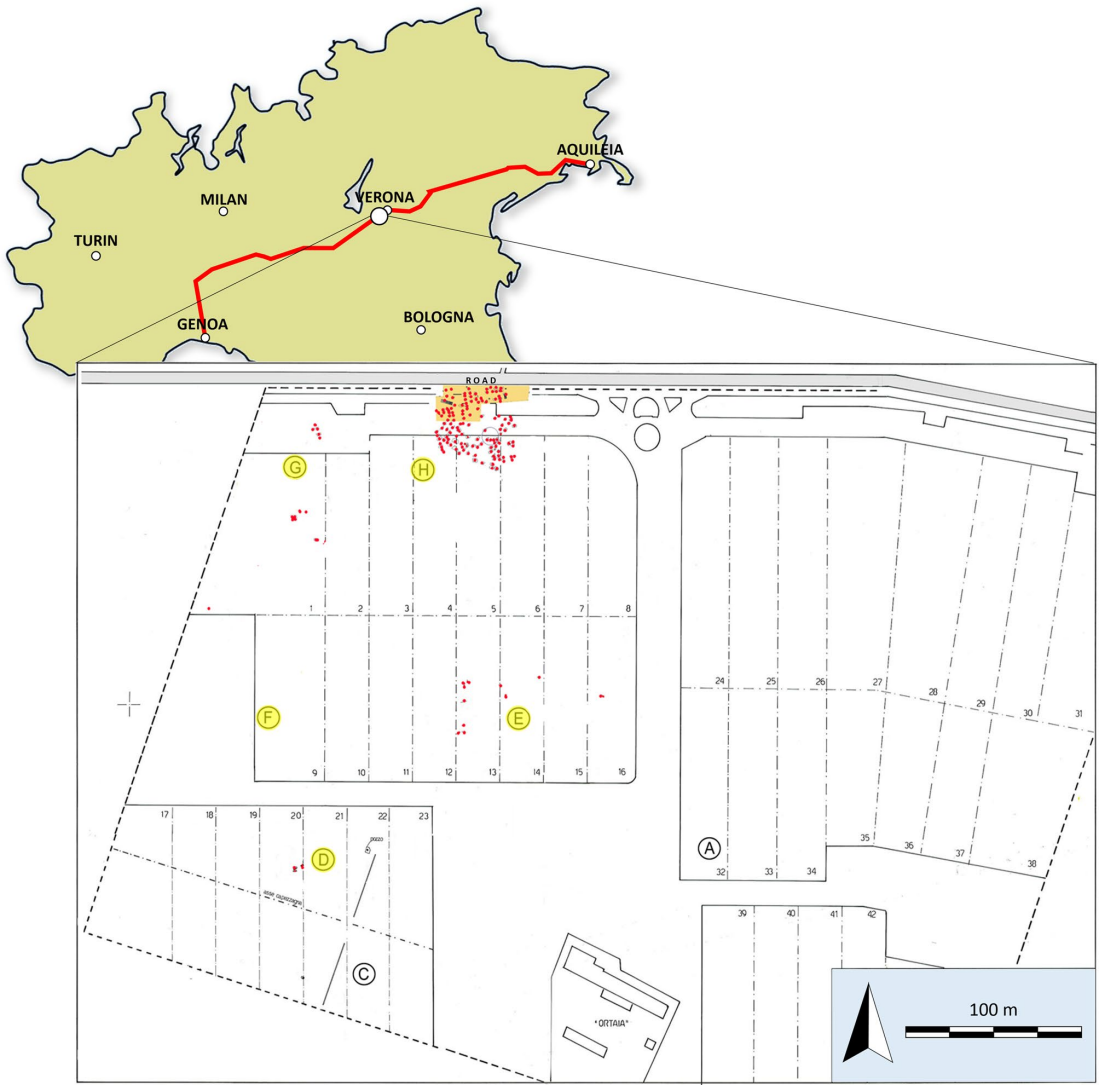
The location of the Longobard cemetery, near Verona (northeastern Italy). The red line represents the ancient Roman road known as Via* Postumia*. Plan of the cemetery with areas of excavations; burials selected for this study come from highlighted areas.

With the two excavation campaigns, an extended necropolis was brought to light, with 164 Longobards burials (Fig. 1).

The cemetery was divided into different sectors: area H is the biggest and represents the core of the cemetery, enclosing the vast majority of the graves; areas D and E only show isolated graves; while area G features pits with multiple burials, a child tomb, three *Grubenhäuser* and traces of a Longobard pit^18^. The graves were dated through typology (i.e., the presence of so-called *Tottenbret*), topography (organization of burials in lines), material culture (i.e., S-shape buckles coming from two burials of subadults, stamped pottery, weapons typology), and ritual features (the burial of an acephalous horse), leading the archaeologists to suggest that the cemetery of Povegliano Veronese was one of the earliest after the settlements of Longobards following the arrival of king Alboin in Italy^17^. Hence, it was possible to bracket the cemetery between the end of the sixth century and the beginning of the eighth century CE. This necropolis was used little more than one and a half century and archaeologists subdivided this period into three intervals^17^: phase 1: 570–620 CE, roughly corresponding to the first phase of Longobards settlement in Italy, phase 2: 620–670 CE; and phase 3: 670–720 CE, associated with the following phases of occupation, for which a level of exchange with the local communities is envisaged.

## Results

Results of isotopic analyses are summarized in Table 2 (strontium data) and Table 3 (oxygen data). Statistical analyses were performed using PAST^19^ and SPSS licensed to Sapienza University of Rome.

**Table 2:** Values of strontium isotopes ratios of human and animal samples with standard error.

ID	Material	^87^Sr/^86^Sr	± 2se
**Humans**			
T 2	Enamel	0.708727	0.000010
T 4B	Enamel	0.708771	0.000800
T 7	Enamel	0.708692	0.000700
T 9	Enamel	0.708587	0.000008
T 17	Enamel	0.708423	0.000010
T 23	Enamel	0.711950	0.000010
T 26	Enamel	0.708364	0.000016
T 35	Enamel	0.708762	0.000700
T 42	Enamel	0.709464	0.000009
T 45*	Enamel	0.710734	0.000011
T 86B1	Enamel	0.708809	0.000012
T 86B2	Enamel	0.708699	0.000012
T 86B5	Enamel	0.708790	0.000011
T 96B	Enamel	0.709394	0.000010
T 96C1	Enamel	0.708644	0.000009
T 96C2	Enamel	0.708918	0.000012
T 213	Enamel	0.709535	0.000600
T 332	Enamel	0.708580	0.000700
T 348B	Enamel	0.708840	0.000016
T 349	Enamel	0.708721	0.000022
T 352	Enamel	0.709924	0.000700
T 362	Enamel	0.708776	0.000700
T 364	Enamel	0.709066	0.000700
T 365	Enamel	0.708540	0.000008
T 370	Enamel	0.708990	0.000800
T 376	Enamel	0.708587	0.000700
T 378	Enamel	0.709592	0.000017
T 380	Enamel	0.709278	0.000010
T 380	Bone	0.708433	0.000009
T 382	Enamel	0.708482	0.000600
T 390	Enamel	0.708687	0.000700
T 395	Enamel	0.709341	0.000007
T 405	Enamel	0.708545	0.000012
T 413	Enamel	0.709516	0.000009
T 414	Enamel	0.708981	0.000012
T 426	Enamel	0.712798	0.000009
T 427	Enamel	0.708640	0.000800
T 429	Enamel	0.708612	0.000700
T 430	Enamel	0.708779	0.000600
T 489	Enamel	0.709529	0.000011
T 489	Bone	0.708782	0.000012
**Fauna**			
*Equus caballus*	Enamel	0.70878	0.000021
*Equus caballus*	Bone	0.70925	0.000009
*Bos taurus*	Enamel	0.70943	0.000013
*Ovis vel capra*	Enamel	0.70882	0.000014
*Sus domesticus*	Enamel	0.70896	0.000010
*Sus domesticus*	Bone	0.70866	0.000011
**Soil**			
Soil T 45		0.70883	0.000012
Soil T 348		0.708476	0.000006
Soil T 413		0.708432	0.000012
Soil T 426		0.708719	0.000008

*Excluded from data analysis, as chronologically inconsistent with the Longobard group.

**Table 3:** Stable oxygen isotope data reported as δ^18^O_ph_ of the phosphate group of bioapatite and water.

ID	Material	10^3 18^δ_ph (V-SMOW)_	10^3 18^δ_w_ ± 2.5 _(V-SMOW)_
**Humans**			
T 9	Enamel	17.2	− 6.6
T 9	Bone	16.1	− 8.7
T 23	Enamel	18.6	− 4.0
T 23	Bone	16.5	− 7.9
T 26B	Enamel	19.9	− 1.6
T 26B	Bone	17.6	− 5.9
T 35	Enamel	17.4	− 6.3
T 35	Bone	17.6	− 5.9
T 45*	Enamel	18.5	− 4.2
T 45*	Bone	17.7	− 5.7
T 349	Enamel	18.6	− 4.0
T 349	Bone	16.8	− 7.4
T 352	Enamel	21.1	0.6
T 352	Bone	17.1	− 6.8
T 365	Bone	17.8	− 5.5
T 370	Enamel	18.0	− 5.2
T 370	Bone	17.1	− 6.8
T 380	Enamel	17.5	− 6.1
T 380	Bone	17.9	− 5.3
T 395	Enamel	16.5	− 7.9
T 395	Bone	16.9	− 7.2
T 405	Bone	17.7	− 5.7
T 413	Enamel	16.7	− 7.6
T 413	Bone	17.9	− 5.3
T 426	Bone	17.5	− 6.1
T 430	Enamel	19.7	− 2.0
T 430	Bone	18.2	− 4.8
T 489	Enamel	14.5	− 11.6
T 489	Bone	16.9	− 7.2
Mean		17.6	− 5.8
SD		1.2	2.2
Mean (no outliers)			− 6.0
sd (no outliers)			1.4
**Fauna**			
*Equus caballus-1*	Enamel	15.4	− 10.1
*Bos taurus*	Enamel	16.7	− 8.2
*Bos taurus*	Enamel	15.1	− 9.8
*Sus domesticus*	Enamel	17.4	− 6.2
*Sus domesticus*	Enamel	17.6	− 6.0
*Ovis vel Capra*	Enamel	18.9	− 5.6/-5.9
*Equus caballus-1*	Bone	16.1	− 9.1
*Bos taurus*	Bone	16.2	− 8.7
*Bos taurus*	Bone	19.3	− 5.6
*Sus domesticus*	Bone	17.1	− 6.6
*Sus domesticus*	Bone	17.0	− 6.7
*Ovis vel Capra*	Bone	18.1	− 6.2/-6.8
*Ovis vel Capra*	Bone	17.0	− 6.9/-8.0
*Cervus elaphus*	Bone	17.6	− 7.1

*Excluded from data analysis, as chronologically inconsistent with the Longobard group.

### Strontium isotope ratios

**Figure 2:**
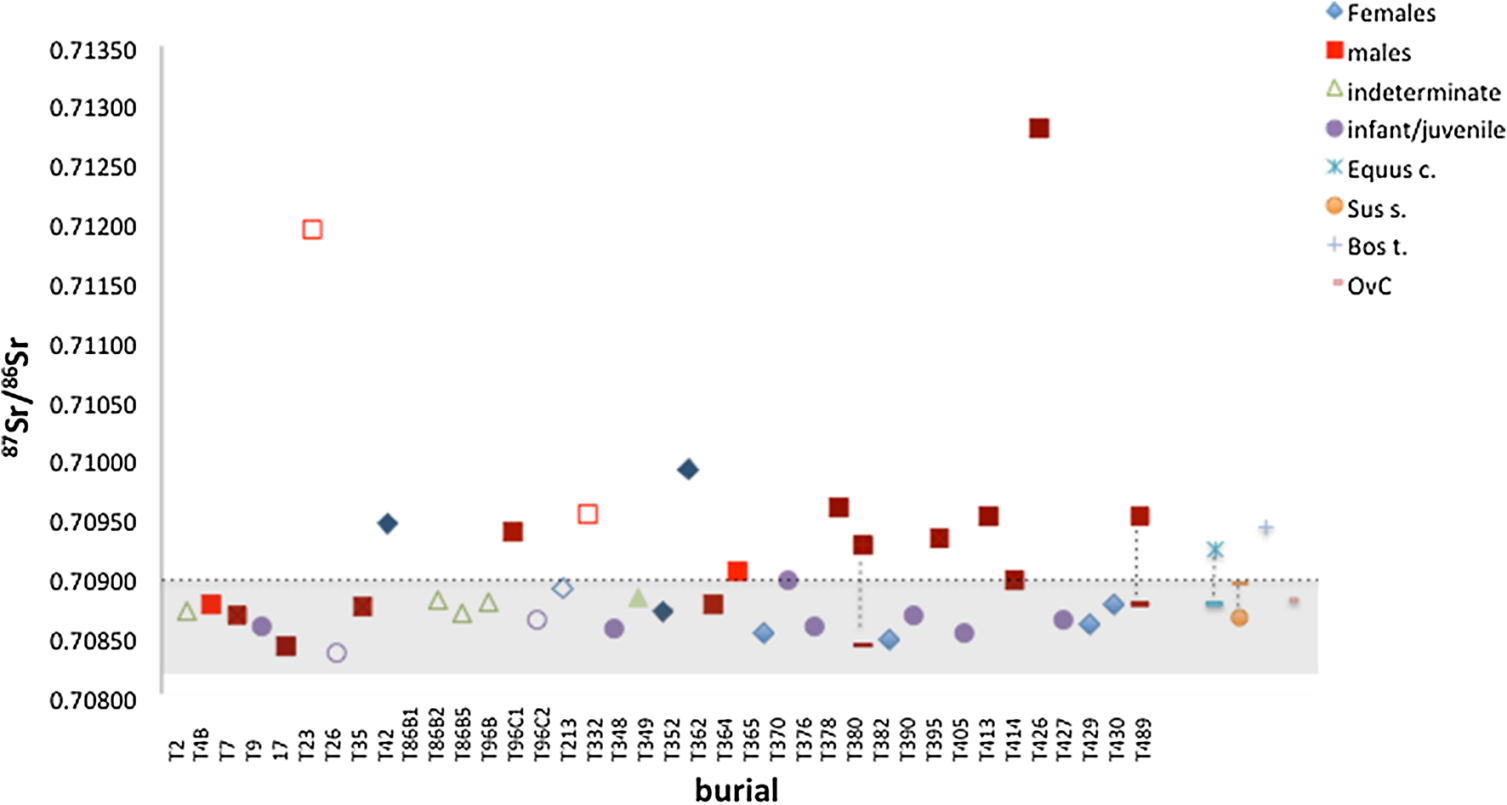
Strontium isotope ratios of human dental enamel and bone and associated fauna. The grey area corresponds to the local Sr range calculated as ± 2sd of the mean soil values^12^. The dashed line represents mean Sr ratio calculated on the faunal samples only. For humans sex and age group (adult, infant, juvenile) are indicated. Individuals linked by the dashed line provide both enamel (line) and bone (maker) data. Filled dark shade = early phase; filled light shade = later phases; empty = no chronology.

Human teeth ratios range from 0.70836 to 0.71280 with a mean of 0.70913 (n = 39), whereas the two human bones ratios are 0.70843 and 0.70878. Mean strontium isotope ratio for the animals is 0.70898 (n = 6) and ranges from 0.70866 to 0.70943 (Fig. 2). One of the individuals analyzed (T 45) later turned out to be of a different cultural horizon and was hence excluded from the analysis. Soil signatures range between 0.70843 and 0.70883 with a mean of 0.70861 (n = 4). Animal tissues fall in the range of the soil samples and of most humans, whereas 19 humans, 1 *Bos taurus* (enamel) and 1 *Equus caballus* (bone) fall outside the range of the analyzed soils.

Cluster analysis of the Sr human teeth data (n = 38) shows three distinct groups, which correspond, presumably, to three different geochemical signatures (Fig. 1 SI). The larger group, consisting of 27 individuals, with the exception of one sample (namely T 364), has a Sr ratio (0.70836–0.70906) that include the ranges of the Povegliano Veronese soil (Table 1 SI). This might represent the portion of the local population, or at least with an origin (given these are dental enamel data) consistent with the Sr signatures of the geological background of the cemetery, which matches exactly the local range defined as ± 2 standard deviations of the mean soil values and consistent with mean values of the animal samples (Fig. 2). The strontium range of this group is also consistent with values measured in modern sediments and human bone and dental enamel of prehistoric populations from neighboring areas^20^ (strontium isotope ratio for the region is discussed in the SI). For simplicity, the individuals of this group are defined “autochthones”. A second group of 9 individuals is more radiogenic (0.70928–0.70992) than the first set; their birthplace, as reflected in the dental enamel, might have been outside the resident isotope range. Hence, they are defined here as “allochthones”. Interestingly, one of the *Bos* enamel and *Equus* bone share the same signature of this group (Table 2), reinforcing the idea of mobility, which might have involved some of the animals investigated (see below). Two further outlying individuals show the highest Sr ratios (0.71073–0.71280) when compared to the other groups. They were coming from localities with largely different ^87^Sr/^86^Sr ratio in the labile portion of soil and, perhaps, ingested water.

### Stable oxygen isotopes

Table 3 shows δ^18^O_ph_ values of the phosphate anion of enamel and bone for all the humans and animal sampled, with the conversion to ingested water δ^18^O_w_ using the equation in Iacumin and Venturelli^21^. The δ^18^O_w_ calculated from the human teeth ranges from − 11.6 to 0.6‰, with a mean of (− 5.1 ± 3.0)‰ (n = 13), while human bones range from − 8.7‰ to − 4.8‰ with a mean of (− 6.4 ± 1.1)‰ (n = 16). Mean δ^18^O_w_ for the animal teeth is − 7.3‰ (n = 6), ranging from − 10.5‰ to − 3.6‰, whereas for animal bones mean δ^18^O_w_ is − 6.4‰ (n = 8) with values ranging from − 8.6‰ to − 2.7‰. Mean δ^18^O_w_ values for both human and animal bones is − 6.4‰, whereas there is a greater gap between the average values of human and animal teeth (− 5.1‰ and − 7.3‰ respectively).

Cluster analysis of human enamel δ^18^O_w_ (n = 13) shows three groups, which are presumed proxies of local water sources (Fig. 2SI,a): the first one (9 samples) shows values that range from −7.9‰ to − 4.0‰; the second group (3 samples) ranges between −2.0‰ and + 0.6‰. Only the first group could match ingested water similar to the present-day precipitation.

Cluster analysis of human bone shows only two groups (Fig. 2 SI, b). The first one ranges from − 8.7 to − 6.8‰ and the other from − 6.1 to − 4.8 ‰.

## Discussion

Because each isotopic parameter serves as a proxy of distinct geological or environmental contexts, strontium and oxygen data are first discussed separately and further in combination.

Wide variation in enamel strontium isotopic composition suggests marked differences in geological background, which probably characterize an area larger than the one investigated (Fig. 2). Hence, individuals either lived in areas with distinct strontium isotope imprinting or ate foods from distinct contexts during enamel formation. Two humans bone signatures have ^87^Sr/^86^Sr values, which are in local soils range.

In order to assess a possible relation between origin of the individuals and time of use of the cemetery, we compared strontium ratios with chronological attribution of the burials. The group of “autochthones” includes individuals with both early and late grave goods. The individuals with earlier grave goods (dated to the second half of the 6th century/early decades of the 7th) could represent the Longobards born in the Povegliano Veronese area (unsurprisingly some of them are subadults) or, rather, local non-Longobards who interacted and mingled with the colonizing group. In both cases, Sr signatures would be consistent with that of the local geology.

The Sr ratio range of the group of “allochthones” suggests that there was some movement among Italian Longobard villagers. There is an alternative to this hypothesis: it is noteworthy that the same Sr range matches with values from the Lake Balaton area in Hungary^3^. This region, called *Pannonia* by the Romans, was the place of settlement of the Longobards during the sixth century CE, immediately before their descent to Italy, hence might represent the place of origin of some of the individuals in the sample. This interpretation is supported by the fact that the group of “allochthones” (with the only exception of T 364) is characterized by early grave goods dated to the first phase of occupation of the cemetery (570–620 CE).

Two individuals (T 380 and T 489), belong to the group of “allochthones”, and are the only ones providing strontium isotope ratios from both teeth and bones. With due caution, given known problems of diagenesis, we could argue that they were not born in the same geological region in which they died, considering different signatures of enamel (outside the local range) and bone (within the local range) (Fig. 2).

Finally, individuals from T 23 and T 426 showed Sr isotopic values of dental enamel that are significantly more radiogenic than those of the rest of the population. Although for only one of the two (T 426) we have a clear chronological attribution (i.e., phase 1), we could argue that such individuals were probably coming from a further geological region, which does not correspond either to that of the “autochthones” or to that of the “allochthones”. T23 was an adult male, buried with no grave goods; despite his alien origin, the man must have mixed with the local group some time prior to his death, so as to justify his presence in the cemetery. T 426 is a male died in later adulthood (40–50 years), found with a funerary assemblage dated to the initial phase of the Longobard settlement in Italy (570–620 AD); like for the allochthones dated to the early phases of occupation, this man suggests an alien provenience although from a more radiogenic geological background.

Most male individuals have isotopic value of Sr outside the local range compared to females, which appear to be statistically significant (Kruskal–Wallis; p_males=females_ = 0.014). This could be proof that men were more likely to move as opposed to women. Interestingly, Sr values distributed according to the age of the individual, show that all the individuals up to the age of 15 have local values, while greater variability is found among older individuals (Fig. 2).

Taking into account the uncertainty on the evaluation of ^18^δw from ^18^δph (± 2.5‰) we can state that the variability around the mean of the ^18^δw values calculated from the ^18^δph of human bones (Table 3) is small (− 6.4 ± 1.1‰). Excluding the two “outliers” cited above, the variability is also low for enamel (− 5.8 ± 1.4‰). Moreover, the central values for enamels and bones have a high probability to be the same (t-test: p_same mean_ = 0.24; Mann–Whitney: p_same mediane_ = 0.38). Thus, we can state that (a) there is no evidence of variation of isotope features of water ingested by humans found at the site, and that (b) most individuals resided in the same place at least for the final phase of their life (given these are bone values). Moreover, if the data are compared with the present-day precipitation (about 7.7‰ ± 2‰ at Verona, a town not far from Povegliano Veronese; Longinelli and Selmo, 2003), we can also state that there is no evidence of a significant climatic difference in respect to today. The results also suggest that humans and animals drank from the same water sources, which should not be surprising, in fact the area is and was characterized by abundant springs.

Once again, cluster analysis performed for dental enamel oxygen values appear to differentiate three sub-groups of individuals (Fig. 2 SI, a). These might be associated with at least three sources of water, which may result from as many geographical areas: the largest group, (n = 9; − 7.9 to − 4.0‰) might come from the Povegliano Veronese area, given that it partially shares mean values associated with the region; a second group, with lighter δ^18^O (− 1.9 to 0.5‰) could be referred to ingestion of abundant evaporated water. One individual, with low δ^18^O (T489; δ^18^O = − 11.6‰), is consistent with a provenance from a colder area; the individual (a non-local for Sr data) is interestingly associated with early Longobard evidence.

The individual T26 is an eight years old child. In this case, the oxygen data is so high not only for the ingestion of more enriched water but also because the tooth analyzed (i.e., M1, that mineralizes during a time range that goes from in utero to the first months of life) registers the enrichment of the mother's milk (“weaning effect”). In fact, it is well known that δ^18^O of human milk is enriched of about 1–2‰ in respect to the drinking water^22^.

**Figure 3:**
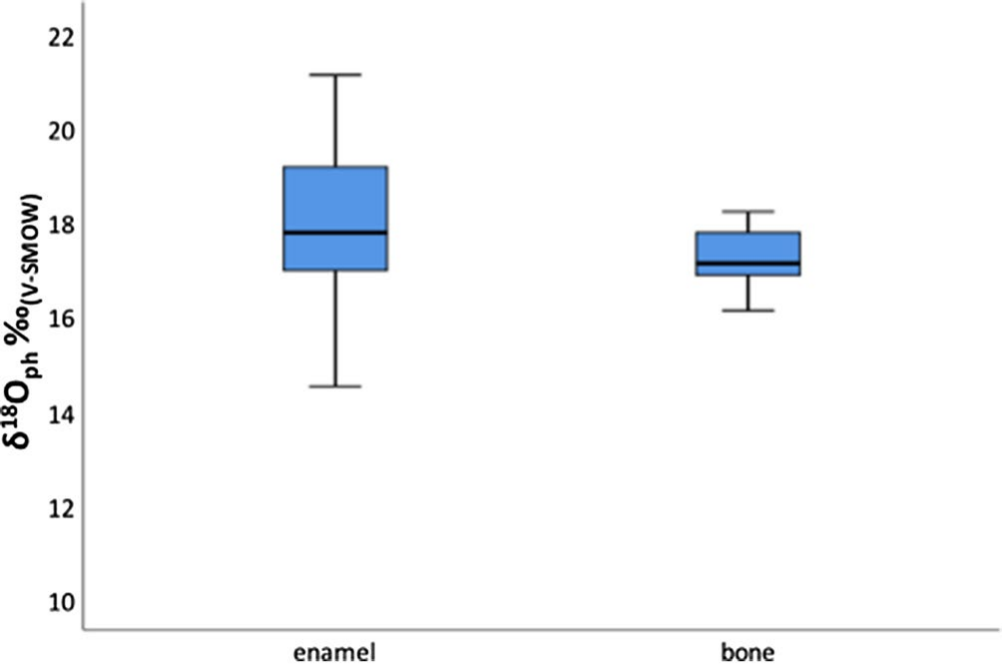
Whisker and box plot of δ^18^O_ph_ in dental enamel and bone for Povegliano Veronese humans.

We could compare the δ^18^O in enamel and corresponding bone for 13 of the sixteen humans examined (Fig. 3). The δ^18^O_w_ of bones appear homogeneously distributed, conversely, large variations are noted in the teeth values. In particular, seven of the thirteen enamel values are quite different from those of the corresponding bone, thus, it is possible to suggest a clear change in the water sources between the first and the last phase of life of such individuals. The widest range is registered in T352 (7.3‰), a non-local (for Sr signature) with grave goods featuring ornaments. Instead, for the other 6 individuals, the difference between bone and teeth values is lower than the normal intra-population variability (about 2‰),this suggests that these individuals during their life drunk water with the same isotopic imprinting.

Interestingly, some individuals changed the water supply between the first and last phases of their life from higher to lower oxygen values or vice versa. Among these, individual T489 has values ranging from − 11.6‰ in enamel to − 7.2‰ in the bone suggesting migration from a colder to a warmer region.

The δ^18^O values for bones do not exhibit variation between the sexes: male data range from − 5.4 to − 7.9‰ with an average of − 6.6‰ while female data range from − 5.3 to − 7.4‰ with an average of − 6.01‰.

As for the enamel values of the animals, one *Bos* and two *Sus* share the same δ^18^w found both in human bones and enamel, suggesting that some humans and animals drank from the same water sources. Different values are the most negative δ^18^w of the *Equus* and a *Bos*, which, probably, drunk from more negative water sources located in a different area. Further, the most positive δ^18^w of one ovicaprid might be indicative of evaporated water source. The animal bone δ^18^w are generally more homogeneous, except for the *Equus* and *Bos* with the more negative values discussed earlier (respectively with − 8.6 and − 8.5‰), and for a *Bos*, with the lighter value of − 2.7‰. For these three latter cases, we could hypothesize an origin at different latitudes from Povegliano Veronese with later movement to the area.

**Figure 4:**
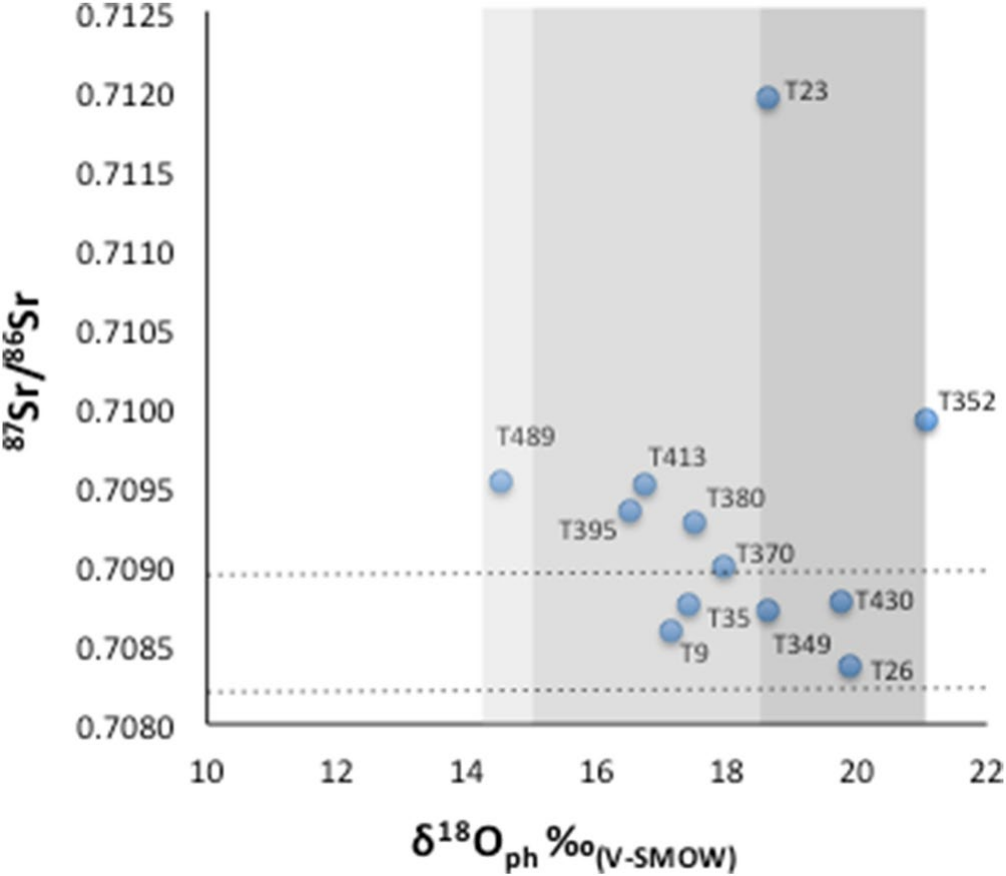
δ^18^O_ph_ and ^87^Sr/^86^Sr of human dental enamel of selected individuals (n = 12). The grey areas roughly correspond to the possible three water sources. Values between the dashed lines correspond the local Sr range calculated as ± 2sd of the mean soil values^12^.

Only 12 of the 38 individuals allowed us to combine ^87^Sr/^86^Sr and δ^18^O (dental enamel) data (Fig. 4). The individuals considered to be “autochthones” according to Sr values, appear to have had two main sources of water: one consistent with the values from Povegliano Veronese and another perhaps with higher isotope values. The “allochthones”, instead, probably drank from three distinct regions: one characterized by negative values, another compatible with Povegliano Veronese area, and the last one compatible with climatic conditions similar to those of Povegliano Veronese, but with different geological substratum. Some individuals show inconsistency in the Sr signature as respect to oxygen ratio: as an example T23 and T349, T430 and T26 are an allochthone and three autochthones respectively, however share similar oxygen values. This apparent inconsistency might be explained if we assume that T23 ingested water from an area with oxygen values consistent with those from Povegliano Veronese.

The results of this study offer significant contribution, which can be summarized as follow:

1. Sr isotope ratios measured for 27 samples from Povegliano Veronese had values (0.70836–0.70906), which indicate that most of the individuals investigated—either of early or later phases—were likely to be local inhabitants. We are keen to suggest such individuals were “autochthonous” to the region and were likely to drink water mostly from the area of Povegliano Veronese.
2. Sr ratios (0.70928–0.70992) for a small (n = 9) “allochthonous” group of individuals match the Sr geochemical signatures from a different region. We cannot rule out movement from nearby regions, however we favor a possible area of origin to be in Hungary^3^, a region known to have been long occupied by the Longobard people. Most of the individuals in this group are dated to the early phases of occupation of the cemetery. For some of these individuals, oxygen values indicate that water ingested came from a source that is not in the area of Povegliano Veronese.
3. An extremely small percentage of Sr values (0.71073–0.71280) hint to a migration of “outliers” that includes movement from a broader European region. This hypothesis appears to be confirmed – at least for one individual – by oxygen values.

Strontium and oxygen data measured in the skeletal tissues of both animal and humans from Povegliano Veronese unveil pattern of residential mobility of the Longobards in Italy. Integration of isotopic data and archaeological evidence at the cemetery seems to suggest that the first generation of Longobard “colonizers” in Italy have likely merged at an early stage with local inhabitants. Further, the second and following generations of Longobards were native to the region and have acquired foods and water from local environments. Despite a mostly “local” provenience, we cannot rule out the influx, across the whole phase of occupation of the cemetery, of alien people either from a wider “Italian” territory or from Central Europe. The results obtained seem to provide evidence of the integration and further “Latinization” of the Longobards in the Mediterranean, which eventually led to the formation of a new Longobard identity in Italy (Rotili, 2010).

## Material and methods

For this study soil, tooth, and bone specimens of humans and animals were sampled for strontium isotope ratios (^87^Sr/^86^Sr), whereas teeth and bones of humans and animals were sampled for stable oxygen isotopes analysis δ(^18^O/^16^O). Sample collection, preparation and analysis was performed in accordance with relevant regulations for the treatment of ancient human remains. Permission to analyze the samples was granted by the local SABAP (Soprintendenza Archeologia, Belle Arti e Paesaggio per le province di Verona, Rovigo e Vicenza).

Because of the bad state of preservation of some of the human teeth, sampling for isotopic study could not be consistent. We decided to use the tooth that was mostly represented. Hence, we preferentially sampled canines, with second and third molars as possible substitutes. The enamel of the canines forms from 4 months to six-seven years of life; whereas the second molar forms from three to seven-eight years and the third molar from seven-eight to more than twelve years representing late childhood-adolescence. For dental enamel sampling, we collected for both analyses approximately 50 mg of enamel powder, with a microdrill mounting a diamond burr, from the lingual surface of the tooth. The extracted enamel came from the lower half of the crown in order to prevent alterations due to metabolism in early formation phases^23^ and to reduce the noise in the data linked to random sampling.

For the analysis of bone, we took approximately 50 mg of cortical bone tissue, mostly from ribs^24^. Several authors recommend to avoid analyzing bone for Sr isotope ratios because it is particularly prone to diagenetic processes^12,25-28^. However, in order to test possible diagenesis, and to observe the difference between tooth value and bone value in the same individual, we run two bone analyses for strontium and sixteen analyses for oxygen.

In order to define the local range at Povegliano Veronese and to compare the data obtained from teeth and bones of humans and animals, we took soil samples from the burials of area H, namely: T 348, T 426, T 413, T 45 (Table 2).

In total, 39 individuals were selected from 35 burials at Povegliano Veronese, while a grand total of 8 animal samples came from a midden excavated near the main concentration of burials and dated to the phase of use of the cemetery (G. De Zuccato pers. comm.). We selected all animal species determined within the midden (using a MNI criterion, Table 2 SI); both domesticated and wild species were available for analyses, namely: one individual of *Equus caballus*, two individuals of *Bos taurus*, two of *Sus domesticus*, two of *Ovis vel Capra* end one of *Cervus elaphus* (Table 1).

The burials were selected according to position within the cemetery, dating of grave goods and typology of tomb structure^17^, we tried to keep a balance in the composition of the sample in accordance to sex and age at death of the individuals. In order to examine strontium and oxygen ratios in humans over time we chose 21 burials dated to phase 1, 9 burials of subsequent phases (2 and 3), and 4 burials with undetermined chronology. We further sampled individuals from 3 multiple burials, which despite not providing a date were considered worthy of investigation.

In particular, the strontium isotopic ratio was measured in 39 enamel samples and 2 human bones, 4 soils samples (from area H), 4 animal teeth (from one *Equus caballus*, one *Bos taurus*, one *Sus domesticus* and one of *Ovis vel Capra respectively*) and 2 animal bones (from one *Equus caballus* and one *Sus domesticus*). Oxygen isotopic ratios were measured in 13 human teeth, 16 human bones, and 5 animal teeth (one specimen of *Equus caballus,* two specimens of *Bos Taurus,* two of *Sus domesticus*, one specimen of *Ovis vel Capra)* and 7 animal bones (one specimen of *Equus caballus,* two specimens of *Bos taurus,* two of *Sus domesticus*, two of *Ovis vel Capra* end one of *Cervus elaphus*) (Table 1).

### Strontium isotope ratio (^87^Sr/^86^Sr)

After cleaning the surface of each tooth by abrasion with a diamond burr, 20–30 mg of enamel powered were extracted and digested in 1 ml concentrated ultrapure HCl. The samples were then evaporated to dryness and redissolved in 2 ml 2 M ultrapure HCl.

For bone analysis, the samples were mechanically cleaned and authigenic carbonates removed with CH3COONH_4_ buffer at pH 5 in an ultrasonic bath. Approximately 5 g of cortical bone were reduced to ashes in a furnace at 800 °C for 10 h and then the material was homogenized in an agate mortar and dissolved with 6 N ultrapure HCl. Once the bone dissolution completed, the samples were evaporated to dryness, redissolved in 2 ml 2 N ultrapure HCl.

Soil samples were leaching with 1 N CH_3_COONH_4_ at neutral pH to obtain the NH4-acetate extract that represents organically bound Sr^29^. The extracts were processed for Sr isotopic analysis following the procedure of bone analysis.

Sr was separated from the matrix onto a preconditioned resin column with 2 mL of AG50W-X12 (200 − 400 mesh) following the procedure of Chao et al.^30^.

Isotopic analyses were carried out at IGAG-CNR c/o Dipartimento di Scienze della Terra, as Sapienza, University of Rome using a FINNIGAN MAT 262RPQ multicollector mass spectrometer with W single filaments in static mode. Sr isotopic fractionations was corrected against ^86^Sr/^88^Sr = 0.1194. During the data acquisition, measured isotopic ratios of NBS 987 Sr standard, resulted as ^87^Sr/^86^Sr = 0.710285 ± 10 (2σ; n = 27). The within-run precision, expressed as 2se (standard errors), was better than 0.000012 for Sr. Total procedural blanks were below 2 ng.

### Stable oxygen isotopes analysis

Stable oxygen isotopes δ(^18^O/^16^O)_ph_ analyses on the phosphate group of teeth and bones of human and animal bioapatite (ph) were carried out at the Stable Isotope Laboratory of the University of Parma.

To analyze the oxygen isotopic composition of the apatite phosphate group of bone and tooth of humans and animals we followed the protocol by Stephan^31^.

The sample treatments were the following: samples reacted with 2.5% NaOCl for 24 h to oxidize organic substances; then, the samples were reacted with 0.125 M of NaOH for 48 h to dissolve humic substances, 2 M HF for 24 h, 2 M KOH and buffered amine solution. The solutions were then warmed at 70 °C for 3 h and filtered to collect the precipitated crystals of Ag_3_PO_4_. The crystals were analyzed by means of TC/EA, thermal conversion-elemental unit on line with a mass spectrometer (IRMS).

According to IUPAC (International Union of Pure and Applied Chemistry), the isotope ratio ^18^O/^16^O is expressed as:

$$ {{\delta}}\left( {^{18}} {\text{O}}/^{16} {\text{O}} \right) = \frac{(^{18} {\text{O}}/^{16} {\text{O}})_{{{\text{sample}}}}}{(^{18} {\text{O}}/^{16} {\text{O}})_{{{\text{V}} - {\text{SMOW}}}} } - 1 = \frac{\left[ {1000 \left( {\frac{{(^{18} {\text{O}}/^{16} {\text{O}})_{{{\text{sample}}}} }}{{(^{18} {\text{O}}/^{16} {\text{O}})_{{{\text{V}} - {\text{SMOW}}}} }}{ }{-}{ }1{ }} \right)} \right]}{1000} = \frac{\text{X}}{1000} = {\text{X}}\;\permil $$

where δ^18^O_sample_ and δ^18^O_V-SMOV_ are the isotopic abundances in the *sample* in analysis and in the reference international standard *V-SMOW* (Vienna Standard Mean Oceanic Water), and ‰ = 1/1,000. The estimated analytical prediction uncertainty for ^18^δ is ≤ 0.35‰. Hereafter, for simplicity, we report δ^18^O in place of δ(^18^O/^16^O).

In order to relate the values δ^18^O_ph_ of the ${\mathrm{P}\mathrm{O}}_{4}^{3-}$ anionic group of enamel and bone bioapatite to that, δ^18^O_w_, of the drinking water, we used the following equations:

for humans:

δ^18^O_w_ = 1.847 δ^18^O_ph_ − 0.0384 Iacumin and Venturelli ^21^

for *Capra*:

δ^18^O_w_ = 1.14 δ^18^O_ph_ − 0.0274 Delgado Huertas et al.^32^

for *Ovis:*

δ^18^O_w_ = 0.676 δ^18^O_ph_ − 0.0184 Delgado Huertas et al.^33^

for *Bos*:

δ^18^O_w_ = 0.990 δ^18^O_ph_ − 0.0247 Delgado Huertas et al.^33^

for *Equus:*

δ^18^O_w_ = 1.41 δ^18^O_ph_ − 0.0318 Delgado Huertas et al.^33^

for *Cervus*:

δ^18^O_w_ = 0.885 δ^18^O_ph_ − 0.0227 D'Angela and Longinelli^34^

δ^18^O_w_ = 1.16 δ^18^O_ph_ − 0.0264 Longinelli^13^

(It is noteworthy that the equations used for animals could not to be statistically different one from the other).

## Supplementary information

Supplementary file1 (DOCX 311 kb)
