# Supplementary material for: Strontium and oxygen isotopes as indicators of Longobards mobility in Italy: an investigation at Povegliano Veronese
Source: Sci Rep. 2020 Jul 15;10:11678. doi: 10.1038/s41598-020-67480-x (PMC7363922; doi:10.1038/s41598-020-67480-x)
Supplement: Supplementary file 1 — Supplementary file1 (DOCX 311 kb) [file 41598_2020_67480_MOESM1_ESM.docx]

**Strontium and Oxygen Isotopes as indicators of Longobards mobility in Italy: an investigation at Povegliano Veronese**

Guendalina Francisci^1, 2¶^ Ileana Micarelli^1¶^, Paola Iacumin^3^, Francesca Castorina^4^, Fabio Di Vincenzo^1,2^, Martina Di Matteo^5^, Caterina Giostra^6^, Giorgio Manzi^1^, Mary Anne Tafuri^1^*

**Supplementary Information**

*Reconstructing mobility through strontium and oxygen isotope analysis*

Strontium (Sr) and oxygen (O) isotopic data obtained from human skeletal remains can be used to investigate questions relating to human origins, migration, and diet ^1^. The "isotopic signatures" of strontium (Sr) and oxygen (O) contained in food and drinking water are incorporated, through their intake, in the skeletal tissues (bone and tooth hydroxyapatite) of consumers and reflect the characterization of the places of residence of individuals at the time of tissue formation or remodeling. Strontium and oxygen are the two chemical elements that through their independent isotopic systems (^87^Sr/^86^Sr and ^18^O/^16^O) reflect local geology and climate respectively, hence becoming ecological markers and providing a direct reflection of the place of residence during childhood (if measured in the teeth) and adult life (if measured in the bones) of individuals.

In particular, strontium is an alkaline earth metal, which substitutes for calcium in the food chain, and is introduced in the skeletal tissue. The Sr isotopic ratio measured as ^87^Sr/^86^Sr varies across the landscape according to the age and original rubidium (Rb) content. Because, strontium is relatively heavy, there is no measurable isotopic fractionation inside environmental processes, so it is taken up by organisms, with the local isotopic signature. The two main limitations in the use of strontium isotopes are diagenesis of skeletal tissues linked to the burial environment, and the possibility to reconstruct mobility patterns of ancient human populations only when there is a displacement of residence among territories with different isotopic strontium ratios (i.e., different geological formations) ^2-7^. These limitations may be successfully addressed by careful selection of the tissue, hence by analyzing dental enamel rather than bone ^8,9^, which is less subject to post-mortem alteration, and by using multiple proxies to differentiate among possible geological backgrounds of origin.

Oxygen is a light element that is very abundant in nature. Unlike strontium, several natural processes fractionate O isotopes, such as evaporation of surface waters and precipitation of rainfall ^10,11^. In humans, the oxygen isotopic ratios, δ(^18/16^O), of bone phosphate is controlled by the isotope composition of the drinking water and consequently of meteoric water, but also in part by water contained in food and by the oxygen of the atmosphere ^12-15^. The isotopic composition of precipitation depends on the source water of the precipitation, distance from the coast, altitude, temperature of precipitation and local climate conditions ^15-20^.

As oxygen isotope composition of biophosphate is directly related to that of ingested (drinking) water, using phosphate oxygen- ingested water relations ^21, and references therein^ it is possible to convert δ(^18^O/^16^O) in the phosphate group of bioapatite in δ(^18^O/^16^O) of the ingested water.

The oxygen isotopic composition of bioapatite on animals is used to obtain estimate of the physical conditions of the environment in which they lived, such as temperature, humidity, rainfall but also environmental reservoir, such as drinking water, food and water in food ^12,22^. According to Wright and Schwarcz ^23^, the variation of δ(^18^O/^16^O) within an ancient population is limited to ± 2‰ of δ(^18^O/^16^O) values (see below for the significance of this parameter). Variation wider than 2 ‰ or of 2.5 ‰ ^21^ may be considered as reliable variation that could be due to human geographic movement and dietary heterogeneity.

The preferred tissue for strontium and oxygen isotope analysis of archaeological samples is dental enamel, which is largely resistant to diagenetic change ^24,25^. In fact, during dental enamel formation, which varies depending on tooth type and can reflect a time interval that goes from the intrauterine life to about 16 years of age, the ingested isotopes are incorporated in bioapatite and correspond and reflect the environment/geological imprinting of the area in which the individual was born and raised ^24,26-30^. After enamel's mineralization, the incorporated strontium does not vary, despite drinking water and food subsequently ingested ^31,32^. By contrast, bone apatite records long-term (about the last ten years of life) isotopic signatures because of its continuous turnover during bone mineralization.

*Strontium and Oxygen isotope characteristics of the area*

Povegliano Veronese is located in the Po plain, with altitudes ranging between 35 and 47 m above the sea level. The area is divided into two portions: the northern occupied by the village of Madonna dell'Uva Secca (where the Longobard population settled during their ingression in Italy), and the southern area, delimited by marshes, non-practicable watercourses and numerous natural springs, in a complex water system ^33^. The presence of water was likely a valuable natural resource already during prehistory, but also a problem for land management because of continuous flooding ^34^. This problem ended in 1400 CE when the area was eventually reclaimed ^33,35^. The study area is in the Veneto region (Italy) within a geographic zone dominated by a local to regional geological complexity; for this reason, there is an extremely variable composition of isotopic strontium ratios. As an example, Povegliano Veronese itself lies on sediment dating back to Pliocene period of the RISS glaciation but, because of the alpine and pre-alpine waterways of Quaternary age, which caused continuous fluvial erosion, morainic and alluvial deposits, characterized by a variable granulometry are also present in the area ^36^. In the western part of Veneto, on the border with Lake Garda, there is a high gravelly and calcareous Pleistocene plain. Fifty km northeast of Povegliano Veronese, near the Lessini mountains, begin the Venetian Pre-Alps, characterized by sedimentary and volcanic rocks and by karstic phenomena caused by glacial and wind erosion. Despite recent works on strontium signatures of Bronze Age populations from Veneto ^37,38^ add valuable information on the geochemical background of the area, still little is known. In fact, there are virtually no Sr isotope data published for Povegliano Veronese and in general for Veneto, to produce a comprehensive database, with information available mostly related to wine production in the area. Relevant isotopic data from the literature (from both modern and ancient sites) are summarized in Table 1 SI.

There is a limited literature on oxygen isotope ratios reference values for the area under study and, as known, parameters such as distance from the sea, altitude and latitude, influence the isotopic values of oxygen in a given location. Lécolle ^39^, has determined how the mean δ(^18^O/^16^O) of precipitation decreases from west to east and to a lesser degree from south to north in Western Europe. The same trend is shown also in Italy, but in the northern regions δ^18^O appears to be more uniform ^40,41^. Longinelli and Selmo (2003) published a map of δ(^18^O/^16^O) of modern precipitation, which is only a very rough guide of δ(^18^O/^16^O) for buried individuals, because the mean annual δ(^18^O/^16^O) in local precipitation has likely changed through time.


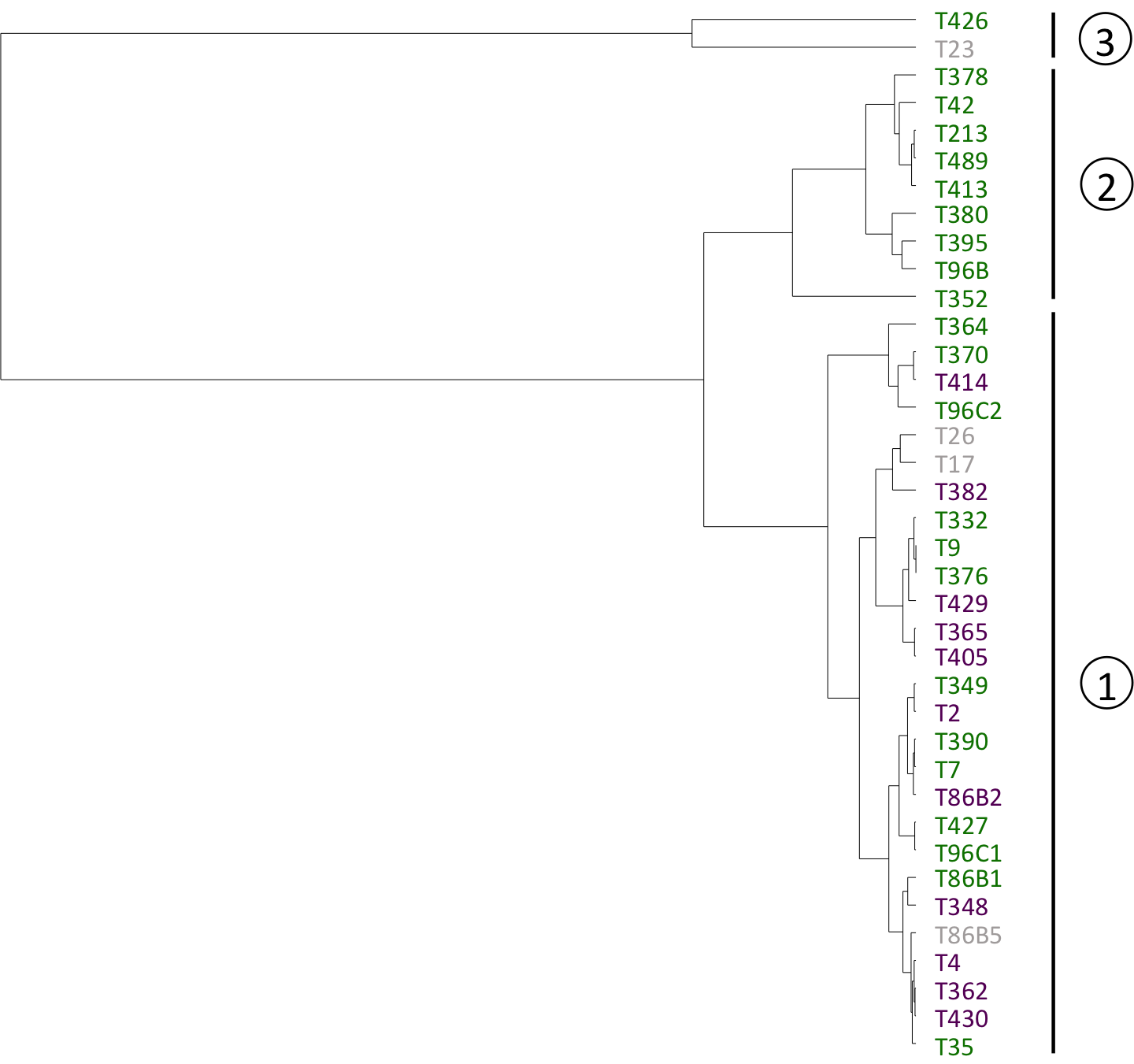


**Figure 1 SI**. Cluster analysis of ^87^Sr/^86^Sr measured in human dental enamel, with the three groups visible. Green = early phase burials; purple = later phase burial; grey = undated graves.


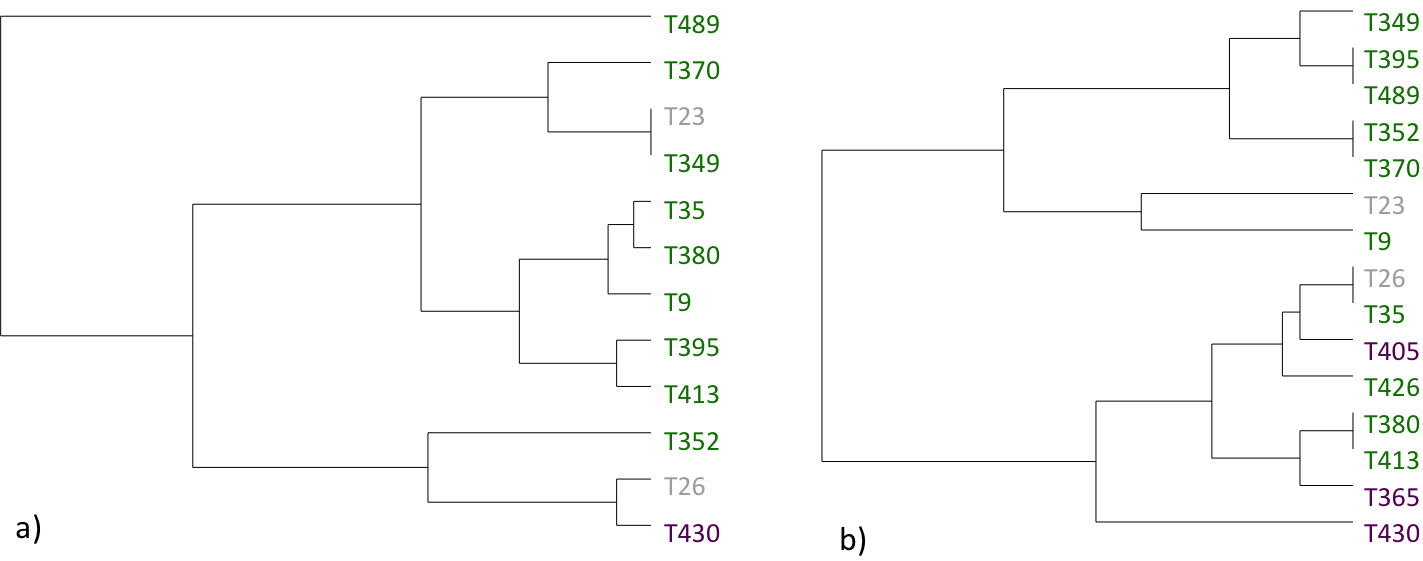


**Figure 2 SI**. Cluster analysis of δ^18^O_ph_ on human dental enamel (a) and bone (b). Green = early phase burials; purple = later phase burial; grey = undated graves.

| **Area of Veneto** | **^87^Sr/^86^Sr range** |
| --- | --- |
| Eastern Veneto ^42^ | 0.7095 – 0.7096 |
| Southern Veneto ^42^ | 0.7089 – 0.7110 |
| Berici mountains ^43^ | 0.7031 – 0.7035 |
| Euganei hills ^43-45^ | 0.7028 – 0.7036,  0.7036 – 0.7037  0.7032 – 0.7036 |
| Lessini mountains ^46^ | 0.7084 – 0.7087 |
| Lower Adige and Lower Brenta Valley^37^ | 0.7089 – 0.7107 |

**Table 1 SI**. Strontium isotope ratios available for different areas of Veneto.

| **Species** | **NISP** | **%** |
| --- | --- | --- |
| Cattle (*Bos taurus*) | 72 | 54.4 |
| Pig (*Sus domesticus*) | 38 | 28.6 |
| Sheep/goats (*Ovis vel Capra*) | 12 | 9 |
| Horse (*Equus caballus*) | 5 | 3.6 |
| Deer (*Cervus elaphus*) | 2 | 1.5 |
| Small-medium equid | 1 | 1.5 |
| Medium mammal | 2 | 0.7 |
| Large mammal | 1 | 0.7 |
| **Total** | **133** | **100** |

**Table 2 SI**. List of identified fauna at Povegliano Veronese.

**References**

1 Faure, G. *Principles of Isotope Geology*. (John Wiley, 1986).

2 Ericson, J. E. Strontium isotope characterization in the study of prehistoric human ecology. *Articolo in rivista* **14**, 503-514, doi:<https://doi.org/10.1016/S0047-2484(85)80029-4> (1985).

3 Montgomery, J., Evans, J. & Chenery, C. Combined lead, strontium and oxygen isotope analysis of the female adult from High Pasture Cave, Isle of Skye. *Uamh an Ard Achadh (High Pasture Cave) and Environs Project, Strath, Isle of Skye*, 101-112 (2004).

4 Bentley, R. A. *et al.* Prehistoric Migration in Europe: Strontium Isotope Analysis of Early Neolithic Skeletons. *Current Anthropology* **43**, 799-804 (2002).

5 Sillen, A., Sealy, J. C. & van der Merwe, N. J. Chemistry and palaeodietary research: no more easy answers. *American Antiquity* **54**, 504-512 (1989).

6 Price, T. D., Grupe, G. & Schröter, P. Migration in the bell beaker period of central Europe. *Antiquity* **72**, 405-411 (1998).

7 Sealy, J., van der Merwe, N. J., Sillen, A., Kruger, F. J. & Krueger, H. W. 87Sr/86Sr as a dietary indicator in modern and archaeological bone. *Journal of Archaeological Science* **18**, 399-416 (1991).

8 Budd, P., Montgomery, J., Barreiro, B. & Thomas, R. G. Differential diagenesis of strontium in archaeological human dental tissues. *Applied Geochemistry* **15**, 687-694, doi:<https://doi.org/10.1016/S0883-2927(99)00069-4> (2000).

9 Trickett, M. A., Budd, P., Montgomery, J. & Evans, J. An assessment of solubility profiling as a decontamination procedure for the 87Sr/86Sr analysis of archaeological human skeletal tissue. *Applied Geochemistry* **18**, 653-658, doi:<https://doi.org/10.1016/S0883-2927(02)00181-6> (2003).

10 Graig, H. & Gordon, L. in *Stable isotope in Oceanographic studies and Paleotemperatures* (ed E. Tongiorgi) 9-130 (Laboratoria di Geologia Nucleare, 1964).

11 Dansdgard, W. Stable isotopes in precipitation. *Tellus* **16**, 436-468 (1964).

12 Longinelli, A. Oxygen isotopes in mammal bone phosphate: A new tool for paleohydrological and paleoclimatological research? *Geochimica et Cosmochimica Acta* **48**, 385-390, doi:10.1016/0016-7037(84)90259-x (1984).

13 Daux, V. *et al.* Oxygen isotope fractionation between human phosphate and water revisited. *Articolo in rivista* **55**, 1138-1147, doi:10.1016/j.jhevol.2008.06.006 (2008).

14 White, C. D., Spence, M. W., Stuart-Williams, H. L. Q. & Schwarcz, H. P. Oxygen isotopes and the identification of geographical origins : the valley of Oaxaca versus the valley of Mexico. *Journal of Archaeological Science* **25**, 643-655 (1998).

15 Luz, B., Kolodny, Y. & Horowitz, M. Fractionation of oxygen isotopes between mammalian bone-phosphate and environmental drinking water. *Geochimica et Cosmochimica Acta* **48**, 1689-1693 (1984).

16 Longinelli, A. & Nuti, S. Revised phosphate-water isotopic temperature scale. *Earth and Planetary Science Letters* **19**, 373-376 (1973).

17 D’Angela, D. & Longinelli, A. Oxygen isotopes in living mammal's bone phosphate: further results. *Chemical Geology: Isotope Geoscience section* **86**, 75-82 (1990).

18 Darling, W. G., Bath, A. H., Gibson, J. J. & Rosanski, K. in *Isotopes in paleaeoenvironmental research* (ed M. J. Leng) 1-66 (Springer, 2006).

19 Yurtsever, Y. & Gat, J. R. Stable isotope hydrology: deuterium and oxygen-18 in the water cycle. *Atmospheric waters*, 103-142 (1981).

20 Longinelli, A. Ratios of oxygen-18: oxygen-16 in phosphate and carbonate from living and fossil marine organisms. *Nature* **211**, 923 (1966).

21 Iacumin, P. & Venturelli, G. The δ18O of phosphate of ancient human biogenic apatite can really be used for quantitative paleoclimate reconstruction? . *European Scientific Journal* **11**, 221-235 (2015).

22 Iacumin, P., Di Matteo, A., Usai, D., Salvatori, S. & Venturelli, G. Stable isotope study on ancient populations of central sudan: Insights on their diet and environment. *American journal of physical anthropology* **160**, 498-518 (2016).

23 Wright, L. E. & Schwarcz, H. P. Correspondence between stable carbon, oxygen and nitogen isotopes in human tooth enamel and dentine : infant diets at Kaminaljuyù. *Journal of Archaeological Science* **26**, 1159-1170 (1999).

24 Hillson, S. *Dental anthropology*. (Cambridge University Press, 1996).

25 Hoppe, K. A., Koch, P. L. & Furutani, T. T. Assessing the preservation of biogenic strontium in fossil bones and tooth enamel. *International Journal of Osteoarchaeology* **13**, 20-28, doi:10.1002/oa.663 (2003).

26 Bryant, J. D., Luz, B. & Froelich, P. N. Oxygen isotopic composition of fossil horse tooth phosphate as a record of continental paleoclimate. *Palaeogeography, Palaeoclimatology, Palaeoecology* **107**, 303-316 (1994).

27 Balasse, M., Smith, A. B., Ambrose, S. H. & Leigh, S. R. Determining sheep birth seasonality by analysis of tooth enamel oxygen isotope ratios: the Late Stone Age site of Kasteelberg (South Africa). *Journal of Archaeological Science* **30**, 205-215 (2002).

28 Balasse, M., Ambrose, S. H., Smith, A. B. & Price, T. D. The seasonal mobility model for prehistoric herders in the south-western Cape of South Africa assessed by isotopic analysis of sheep tooth enamel. *Journal of Archaeological Science* **29**, 917-932 (2002).

29 Bocherens, H., Mashkour, M., Billiou, D., Pellé, E. & Mariotti, A. A new approach for studying prehistoric herd management in arid areas: intra-tooth isotopic analyses of archaeological caprine from Iran / Une nouvelle approche pour l'étude de la gestion préhistorique des troupeaux en zones arides : analyses isotopiques intra-dentaires de caprinés archéologiques d'Iran. *Comptes Rendus de l'Académie des Sciences (2a)* **332**, 67-74 (2001).

30 Sharp, Z. & Cerling, T. Fossil isotope records of seasonal climate and ecology: straight from the horse's mouth. *Geology* **26**, 219-222 (1998).

31 Boaz, N. T. & Hampel, J. Strontium content of fossil tooth enamel and diet in early hominids. *Journal of Paleontology* **52**, 928-933 (1978).

32 Gage, J. P., Francis, M. J. & Triffitt, J. T. *Collagen and dental matrices*. (Butterworth-Heinemann, 1989).

33 Bruno, B. & Giostra, C. in *VI Congresso Nazionale di Archeologia Medievale (L’Aquila, 12-15 settembre 2012).* 216-222.

34 Rottoli, M. in *Longobardi, un popolo che cambia la storia* (eds G.P. Brogiolo, F. Marazzi, & C. Giostra) 158-161 (Schira, 2017).

35 Castiglioni, G. & Pellegrini, G. Illustrative Notes of the Geomorphological Map of the Po plain. *Geografia Fisica e Dinamica Quaternaria* **7**, 207 (2001).

36 Carraro, F., Malaroda, R., Piccoli, G., Sturani, C. & Venzo, S. (Peschiera del Garda, 1969).

37 Cavazzuti, C. *et al.* Mobile elites at Frattesina: flows of people in a Late Bronze Age “port of trade” in Northern Italy investigated through strontium isotope analysis. *Antiquity.* (2019).

38 Cavazzuti, C. *et al.* Flows of people in villages and large centres in Bronze Age Italy through strontium and oxygen isotopes. *PloS one* **14**, e0209693 (2019).

39 Lécolle, P. The oxygen isotope composition of landsnail shells as a climatic indicator: applications to hydrogeology and paleoclimatology. *Chemical Geology: Isotope Geoscience Section* **58**, 157-181 (1985).

40 Longinelli, A. & Selmo, E. Isotopic composition of precipitation in Italy: a first overall map. *Journal of Hydrology* **270**, 75-88 (2003).

41 Giustini, F., Brilli, M. & Patera, A. Mapping oxygen stable isotopes of precipitation in Italy. *Journal of Hydrology: Regional Studies* **8**, 162-181 (2016).

42 Petrini, R. *et al.* Geochemistry and isotope geochemistry of the Monfalcone thermal waters (northern Italy): inference on the deep geothermal reservoir. *Hydrogeology Journal* **21**, 1275-1287 (2013).

43 Macera, P. *et al.* Geodynamic implications of deep mantle upwelling in the source of Tertiary volcanics from the Veneto region (South-Eastern Alps). *Journal of Geodynamics* **36**, 563-590 (2003).

44 Barbieri, M. *et al.* in *Stable isotopes in the Earth Sciences* (1978).

45 Milanini, J.-L. *Approche de la découpe du cadavre dans quelques sépultures préhistoriques d'Ardèche. L'aven Jacques (Lussas), la Grotte des Ailes (Casteljau) et la grotte de Chirolong (Gras)*, Université de Provence - U.F.R. Civilisations et Humanité - Département d'Histoire, (1999).

46 Spencer-Cervato, C. & Mullis, J. Chemical study of tectonically controlled hydrothermal dolomitization: an example from the Lessini Mountains, Italy. *Geologische Rundschau* **81**, 347-370 (1992).
